# Supplementary figures and images for: Immune complex disease in a chronic monkey study with a humanised, therapeutic antibody against CCL20 is associated with complement-containing drug aggregates
Source: PLoS One. 2020 Apr 23;15(4):e0231655. doi: 10.1371/journal.pone.0231655 (PMC7180069; doi:10.1371/journal.pone.0231655)

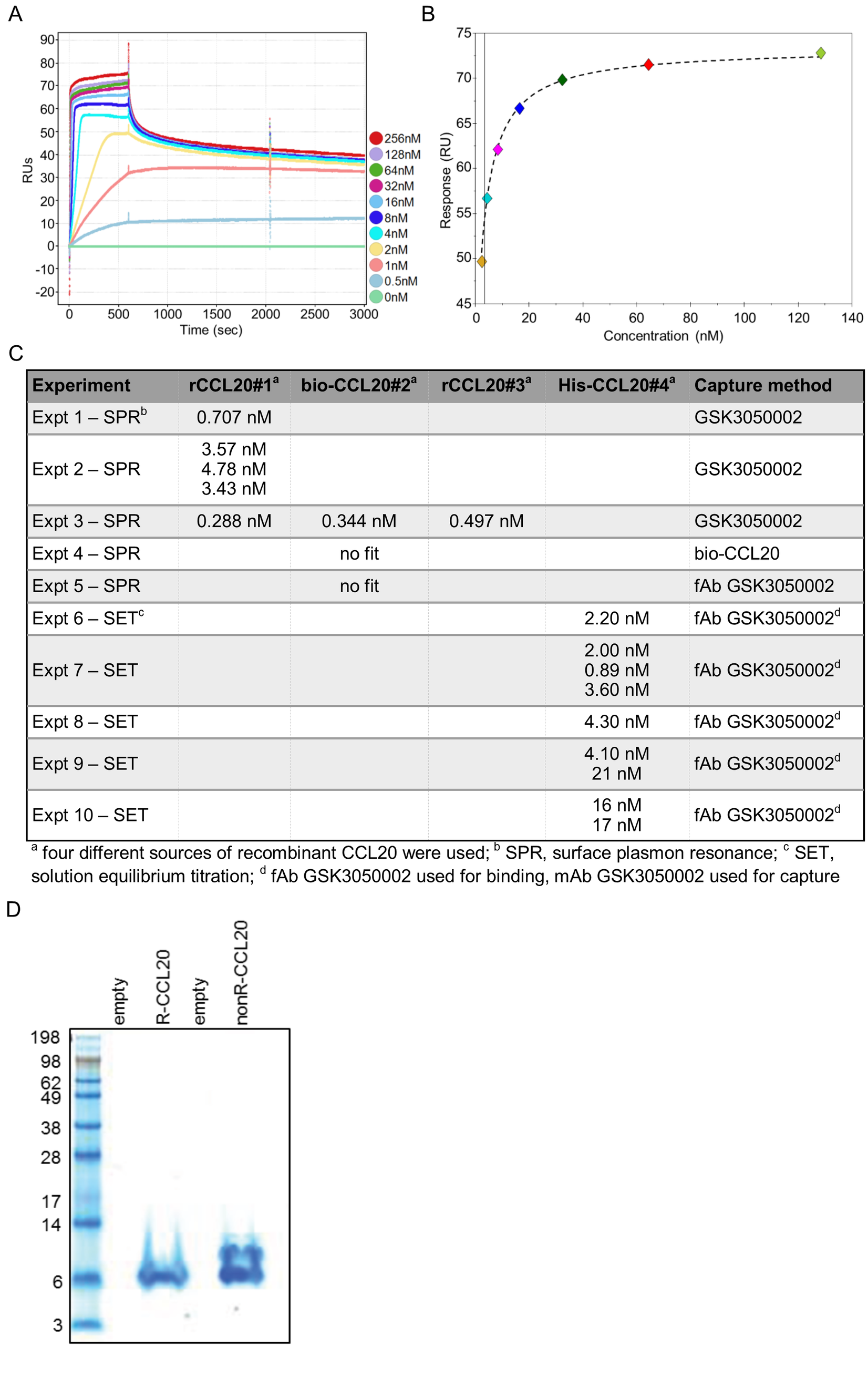

Supplement: S1 Fig — (TIF) [file pone.0231655.s001.tif]

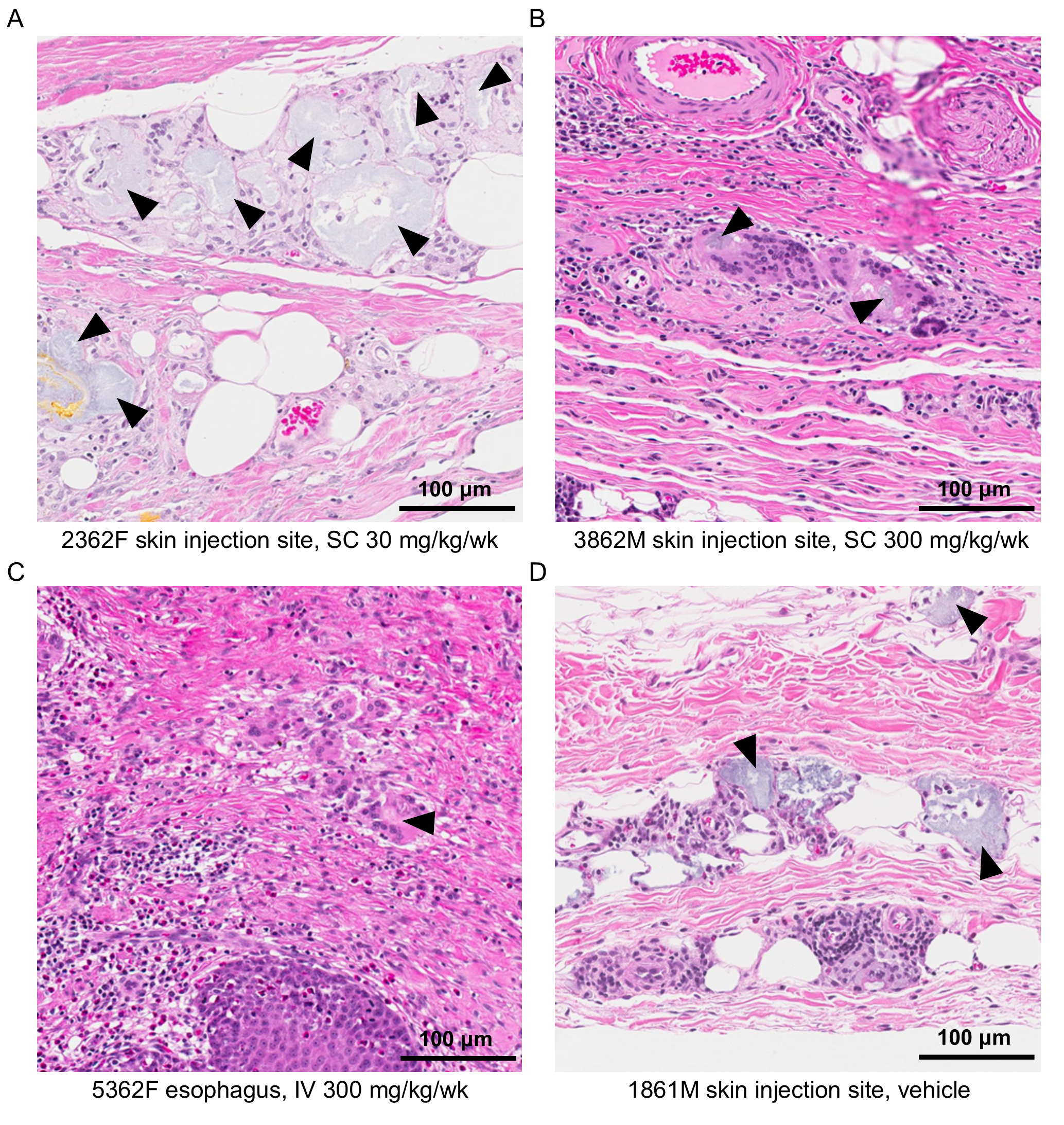

Supplement: S2 Fig — (TIF) [file pone.0231655.s002.tif]
