# Supplementary material for: Immune complex disease in a chronic monkey study with a humanised, therapeutic antibody against CCL20 is associated with complement-containing drug aggregates
Source: PLoS One. 2020 Apr 23;15(4):e0231655. doi: 10.1371/journal.pone.0231655 (PMC7180069; doi:10.1371/journal.pone.0231655)
Supplement: S1 Raw Images — (PDF) [file pone.0231655.s003.pdf]

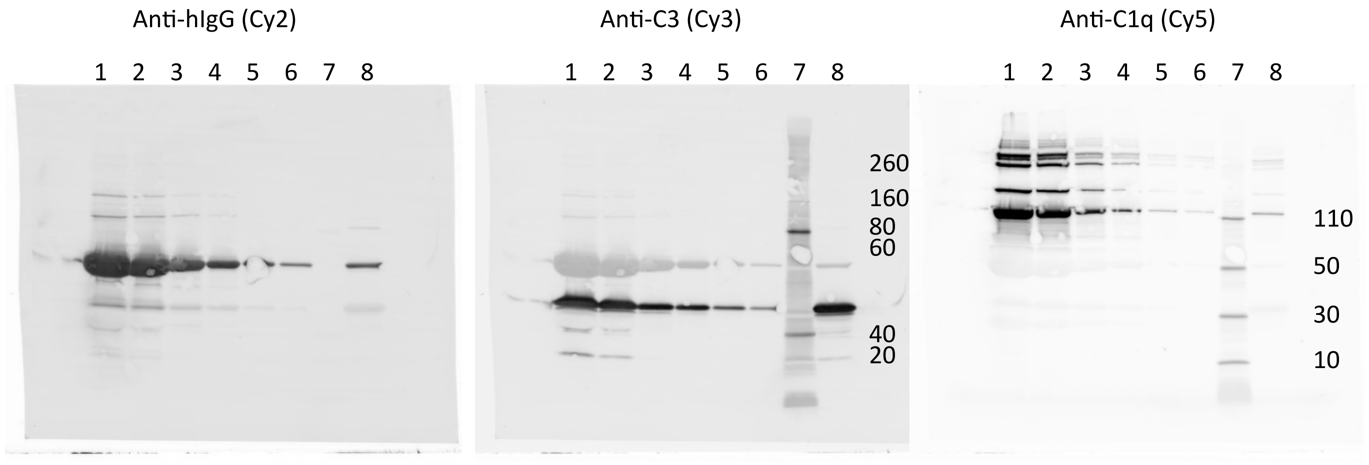

Lane	1	2	3	4	5	6	7	8
Protein	Standard (ng)						marker	sample
hIgG	5000	1000	500	100	50	10		
C1q	500	100	50	10	5	1		
C3	1000	500	100	50	10	2		

Blot images corresponding to Fig3C

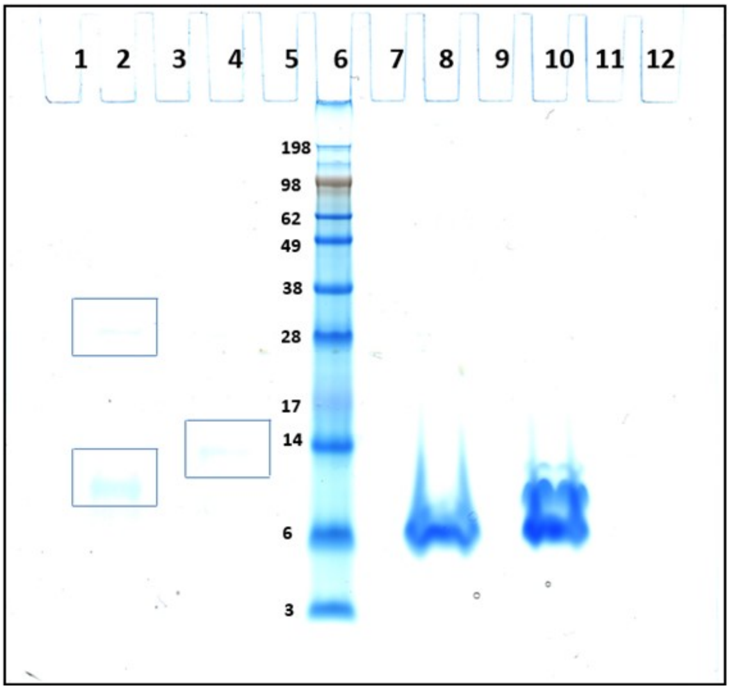

Blot image corresponding to S1D Fig
