# Supplementary material for: Immune complex disease in a chronic monkey study with a humanised, therapeutic antibody against CCL20 is associated with complement-containing drug aggregates
Source: PLoS One. 2020 Apr 23;15(4):e0231655. doi: 10.1371/journal.pone.0231655 (PMC7180069; doi:10.1371/journal.pone.0231655)
Supplement: S1 File — (DOCX) [file pone.0231655.s004.docx]

# Supporting information

# Supplementary materials and methods

## Animals and toxicity study details

Twenty six male and 26 female cynomolgus monkeys obtained from Covance, Alice, Texas, USA were used on the study. The animals were single- or double-housed in stainless steel cages. Compatible pairs, by sex, were housed together per the specifications of USDA Animal Welfare Act (9 CFR Parts 1, 2 and 3). Pairs remained together for group assignment and animals in the same dose group were housed together. If it was not possible to establish or maintain compatible pairs within a group, then animals were housed individually as needed. Pairs were separated (by a removable cage divider) as appropriate during dose administration and/or other study procedures (e.g., urinalysis). Upon the initiation of treatment, the animals were approximately 3 ½ to 5 ½ years old, males weighed 4.5 to 8.8 kg and females weighed 2.4 to 3.9 kg. Animals were provided with environmental enhancement in accordance with the Animal Welfare Standards; Final Rule (9 CFR Part 3) effective March 18, 1991, following the Testing Facility Standard Operating Procedures. Each monkey was offered Certified Primate Diet No. 2050C (Harlan Teklad). All primates were fed twice per day. Food was withheld overnight prior to clinical pathology (including urinalysis) collections and prior to scheduled necropsy. Diets were supplemented with fruits and/or vegetables and treats were presented as necessary or appropriate. Water (supplied and periodically analyzed by New Jersey-American Water Company, Westfield, New Jersey) was available ad libitum except during the period of urine collection.

GSK3050002 was given to cynomolgus monkeys (6/sex at 30 mg/kg/week or 4/sex at 300 mg/kg/week) once weekly for 26 weeks by subcutaneous (SC) injection to the dorsal surface without anaesthesia. GSK3050002 was also given to 2 additional groups of monkeys (6/sex at 30 mg/kg/week or 4/sex at 300 mg/kg/week) once weekly for 26 weeks by slow bolus intravenous (IV) injection to the saphenous or cephalic vein without anaesthesia. A single group of monkeys were given the vehicle (6/sex at 0 mg/kg/week) for 26 weeks by both subcutaneous and intravenous injection. At the end of the treatment period, 4 animals/sex/group were euthanized and necropsied. Remaining animals (2/sex in each of the 30 mg/kg/week groups (SC and IV) as well as control group were held for a 12-week off-dose period. GSK3050002 (Lot Nos. 152388914 and 152388915 [119 mg/mL]) was formulated as a stock solution in 0.80 mg/mL L histidine, 1.1 mg/mL L-histidine monohydrochloride, 60 mg/mL sucrose, 5.3 mg/mL L-arginine hydrochloride, 0.2 mg/mL polysorbate 80, pH 6.0 and administered to cynomolgus monkeys at a dose volume of 3 mL/kg. The following endpoints/parameters were evaluated for all animals: clinical observations including dose site evaluations; body weights; qualitative food consumption; hematology, coagulation, clinical chemistry, and urinalysis; anti-drug antibodies (ADA); immunophenotyping (T, B and T regulatory cells); ophthalmoscopic observations; electrocardiographic evaluations; organ weights; and macroscopic and microscopic observations, including immunohistochemical examination for evidence of immune complex deposits in a subset of animals. Toxicokinetic and ADA evaluation using an acid dissociation assay were performed on samples collected during Weeks 1, 13 and 26. Due to an electrical fire on Days 153 or 156 of the dosing period, staff had to evacuate the Testing Facility for approximately 6 hours; after the event, all animals were bright, alert and responsive. However, because of the site evacuation and some site limitations over subsequent days, the animals missed a 2nd daily feeding and a few observational checks. The pre-treatment, Week 4 and Week 13 residual urine, pre-treatment ADA samples were found to be thawed and the exact temperature for sample storage of the pre-treatment and Week 2 PD samples could not be determined. Environmental monitoring was not available for freezers. These samples were discarded without further analysis.

## Matrix Assisted Laser Desorption/Ionization (MALDI)

Tissue sections (10 μm) of formalin-fixed paraffin-embedded (FFPE) monkey skin were collected in a cryostat at -10 ºC, floated in a water bath at 40 ºC and mounted on glass microscope slides. The slides were then dried in an oven overnight at 37 ºC. The tissue sections were deparaffinised in xylene and then rehydrated by washing 5 min. each in EtOH, 95:5 EtOH:H2O and H2O. Antigen retrieval was performed by incubating the slides in 1mM EDTA at ~95 ºC for 10 minutes. Slides were then washed in 70:30 EtOH:H2O, 95:5 EtOH:H2O and 90:9:1 EtOH:HAc:H2O for 30 seconds each. On-tissue digestion was performed by applying trypsin (0.02 mg/mL) in 50 mM ammonium bicarbonate using a TM Sprayer automated spray device (HTX Technologies). Following digestion, a matrix solution consisting of 2,5-dihydroxybenzoic acid (DHB) (50 mg/mL) in 50:50 methanol: water (1:1, v:v)(0.5% TFA) was applied using a TM Sprayer. Sections (10 µm) serial to those collected for MALDI IMS were collected for hematoxylin and eosin (H&E) staining to correlate ion images with tissue histology. An optical image of each H&E stained serial section was generated using an Aperio ScanScope digital slide scanner (20x and 40x magnifications). Optical, H&E, and ion images were co-registered and arranged into figures using Adobe Photoshop CC software.

## Antibody binding affinity and kinetics

The binding affinity of GSK3050002 to CCL20 was measured using a Biacore™ T200. GSK3050002 was captured onto a CM5 chip, immobilised with protein A/G via primary amine coupling, at different capture levels across three flow cells; flow cell 2 capturing 124 RU, flow cell 3 with 233 RU and flow cell 4 with 462 RU. Human CCL20 (R&D Systems) was passed over the captured antibody at 0nM, 0.5nM, 1nM, 2nM, 4nM, 8nM, 16nM, 32nM, 64nM, 128nM and 256 nM diluted in 1X HBS-EP buffer. The binding curves were recorded and double referenced against a 0nM buffer injection curve and also referenced against the blank flow cell 1. The curves were then fitted to a steady state affinity fit and bivalent analyte model inherent to the Biacore T200 Evaluation software. 50mM sodium hydroxide was used to regenerate the surface after each cycle.

Secondly, binding affinity was measured by capture of biotinylated CCL20 onto immobilised (amine coupling) neutravidin on the surface of a C1 sensor chip. Anti-CCL20 fAb (derived from GSK3050002) was then passed over the captured CCL20 at 128, 64, 32, 16, 8 and 4 nM, binding curves recorded and double referenced against a buffer injection curve and the blank flow cell. The curves were then fitted to 1:1 and steady state binding models using the Biacore T200 evaluation software.

Thirdly, binding affinity was measured by solution equilibrium titration to measure the in-solution affinity of GSK3050002 using the mesoscale discovery (MSD) platform. CCL20 (1.25 or 5 nM) was incubated with serially diluted anti-CCL20 fAb (derived from GSK3050002) starting at 1 μM (11 3-fold dilutions in PBS containing 0.1% IgG-free bovine serum albumin; PBSF). Following a 24 hour incubation, 30µL of anti-CCL20 Ab (GSK3050002) diluted at 20nM or 40nM in PBS was coated on standard bind MSD plates and left to incubate for 30 minutes at room temperature. After coating, washing and blocking of the plates (3 washes with MSD wash buffer (PBSF + 0.05% Tween)), 25µl of the incubated fAb and CCL20 antigen samples were added to the MSD plate, incubated for 2.5 min whilst shaking, then washed once. 25µL of the sulfo-tagged penta-His detection antibody (dilution dependent on batch and signal test read outs) was added and incubated for 3 minutes whilst shaking. The plates were then washed 3 times in MSD wash buffer and then read after addition of 150µL of MSD read buffer T on the MSD sector 6000 imager.

## SDS PAGE analysis

Human CCL20 (Peprotech Cat # 300-29A; Batch - 0609102) was assessed for its purity using SDS-PAGE. 50 mL of 20X NuPAGE® MES or MOPS SDS Running Buffer was added to 950mL of deionised water to prepare 1X SDS Running Buffer. For reduced samples, DTT was added to the sample. 4µg of CCL20 antigen was added to the SDS-PAGE well, which was run under electric current for 30 minutes at 200V. After the gel run finished, the precast gel was separated from its cassette and placed in a square petri dish. Gel was stained with instant blue until band appeared. Gel was washed with water to remove the background stain.

# Supplementary Results

## Routine toxicology endpoints

No GSK3050002-related effects were noted on body weight, food consumption, mortality, ocular abnormalities, coagulation, urinalysis or organ weight. There were no changes in percentage or absolute number of T cells, T cell subsets, B cells or CD4^+^ regulatory T cells. Routine clinical observations did not reveal any GSK3050002-related dermal irritation at either the SC or IV injections sites.

After 26 weeks of treatment, macroscopic observations were confined to skin and subcutis (both separate from and at the SC injection sites) of females given 300 mg/kg/week. Non-injection site lesions included scabbing of the dorsocaudal skin in one female given 300 mg/kg/week SC and hair loss of the dorsocaudal skin and skin of the head in one female given 300 mg/kg/week IV. Dark red areas in the subcutis at the SC injection site were observed in one female given 300 mg/kg/week.

## Supplementary figure legends

**S1 Fig. Analysis of binding affinity**

Representative (A) SPR analysis showed binding of GSK3050002 to CCL20 is biphasic and does not fit the 1:1 binding model. Steady state analysis (B) provides a better model to fit binding data. (C) Overview of all binding experiments performed. (D) SDS PAGE analysis of recombinant CCL20 under reducing (R) and non-reducing (nonR) conditions shows recombinant CCL20 can form weak dimers. Representative data in (A-B) are from Expt 2 in (C).

**S2 Fig. Findings of amorphous‑to‑crystalline material following chronic dosing with GSK3050002**

Representative H&E images of amorphous‑to‑crystalline material at the subcutaneous injection site in skin (A-B, D) and in the esophagus after IV dosing (C). Examples of extracellular (A, D) and intracellular (B, C) are shown. F = female, M = male, numbers indicate animal identification.

## Supplementary tables

**S1 Table. Gender-Averaged Toxicokinetic Parameters for GSK3050002 from Male and Female Cynomolgus Monkeys Following Subcutaneous or Intravenous (Slow Bolus) Administration of GSK3050002**

| **Parameter** | **Period** |  | **Subcutaneous dose of GSK3050002 (mg/kg/wk)** | | **Intravenous dose of GSK3050002 (mg/kg/wk)** | |
| --- | --- | --- | --- | --- | --- | --- |
|  |  |  | **30 (n=12)** | **300 (n=8)** | **30 (n=12)** | **300 (n=8)** |
| AUC_0-168_  (mg.h / mL) | Week 1 | **Mean** | **30.0** | **187** | **42.2** | **239** |
|  |  | Min | 25.4 | 117 | 37.4 | 160 |
|  |  | Max | 39.9 | 226 | 47.0 | 345 |
|  | Week 13 | **Mean** | **60.1^a^** | **252** | **54.3** | **306** |
|  |  | Min | 37.6^a^ | 179 | 26.8 | 178 |
|  |  | Max | 95.0^a^ | 338 | 65.8 | 397 |
|  | Week 26 | **Mean** | **64.4^a^** | **236^b^** | **55.8^c^** | **311** |
|  |  | Min | 38.2^a^ | 155^b^ | 43.1^c^ | 188 |
|  |  | Max | 105^a^ | 283^b^ | 66.6^c^ | 401 |
| C_max_  (mg/mL) | Week 1 | **Mean** | **0.286** | **2.21** | **0.819** | **7.23** |
|  |  | Min | 0.219 | 1.34 | 0.671 | 6.06 |
|  |  | Max | 0.390 | 3.15 | 0.954 | 9.54 |
|  | Week 13 | **Mean** | **0.517^a^** | **2.35** | **0.912** | **8.17** |
|  |  | Min | 0.363^a^ | 2.09 | 0.760 | 6.31 |
|  |  | Max | 0.736^a^ | 2.82 | 1.05 | 10.6 |
|  | Week 26 | **Mean** | **0.551^a^** | **2.27^b^** | **0.883^c^** | **8.44** |
|  |  | Min | 0.343^a^ | 1.77^b^ | 0.747^c^ | 6.62 |
|  |  | Max | 0.888^a^ | 2.83^b^ | 1.11^c^ | 10.5 |

^a^ n=11; One female was excluded from mean/median toxicokinetic parameter calculations due to overall lower concentrations across entire profile, ^b^ n=7, One female was excluded from mean/median toxicokinetic parameter calculations due to sharp decline in serum concentrations, ^c^ n=10, One male and one female were excluded from mean/median toxicokinetic parameter calculations due to sharp decline in serum concentrations

**S2 Table. Summary of histopathological intracellular and extracellular amorphous to crystalline material findings**

|  | Males | | | | Females | | | |
| --- | --- | --- | --- | --- | --- | --- | --- | --- |
| Group | Animal # | IHC results | Tissue | Affected injection sites | Animal # | IHC results | Tissue | Affected injection sites |
| Vehicle  (IV and SC) | 1360 | -- |  |  | 1860 | -- |  |  |
|  | 1361 | -- |  |  | 1861 | Extracellular material | Skin (injection site) | 1/4 |
|  | 1362 | Intracellular material | Skin (injection site) | 2/4 | 1862 | -- |  |  |
|  | 1363 | -- |  |  | 1863 | -- |  |  |
|  | 1364-R | -- |  |  | 1864-R | -- |  |  |
|  | 1365-R | -- |  |  | 1865-R | -- |  |  |
| 30 mg/kg/week SC | 2360 | -- |  |  | 2860 | -- |  |  |
|  | 2361 | Intracellular material | Skin (injection site) | 1/4 | 2861 | Intracellular material | Skin (injection site) | 1/4 |
|  | 2362 | Extracellular material | Skin (injection site) | 3/4 | 2862 | -- |  |  |
|  | 2363 | -- |  |  | 2863 | -- |  |  |
|  | 2364-R | -- |  |  | 2864-R | -- |  |  |
|  | 2365-R | -- |  |  | 2865-R | -- |  |  |
| 300 mg/kg/week SC | 3360 | Intracellular material | Skin (injection site) | 3/4 | 3860 | Intracellular material | Skin (injection site) | 1/4 |
|  | 3361 | Intracellular material | Skin (injection site) | 2/4 | 3861 | Intracellular material | Skin (injection site) | 1/4 |
|  | 3362 | Intracellular material | Skin (injection site) | 1/4 | 3862 | Intracellular material | Skin (injection site) | 2/4 |
|  | 3363 | Intracellular material | Skin (injection site) | 2/4 | 3863 | Intracellular material | Skin (injection site) | 1/4 |
| 30 mg/kg/week IV | 4360 | -- |  |  | 4860 | -- |  |  |
|  | 4361 | -- |  |  | 4861 | -- |  |  |
|  | 4362 | -- |  |  | 4862 | -- |  |  |
|  | 4363 | -- |  |  | 4863 | -- |  |  |
|  | 4364-R | -- |  |  | 4864-R | -- |  |  |
|  | 4365-R | -- |  |  | 4865-R | -- |  |  |
| 300 mg/kg/week IV | 5360 | -- |  |  | 5860 | Intracellular material | Liver | NA |
|  | 5361 | -- |  |  | 5861 | -- |  |  |
|  | 5362 | Intracellular material | Esophagus | NA | 5862 | -- |  |  |
|  | 5363 | -- |  |  | 5863 | Intracellular material | Kidney | NA |

IHC = immunohistochemistry; R (shaded) = off-dose animal; -- = no findings; NA = Not Applicable as only 1 tissue sample from these animals was examined.
