# Supplementary material for: Immune complex disease in a chronic monkey study with a humanised, therapeutic antibody against CCL20 is associated with complement-containing drug aggregates
Source: PLoS One. 2020 Apr 23;15(4):e0231655. doi: 10.1371/journal.pone.0231655 (PMC7180069; doi:10.1371/journal.pone.0231655)
Supplement: S1 Table — (DOCX) [file pone.0231655.s005.docx]

## Supplementary Tables

Supplementary Table S1. Gender-Averaged Toxicokinetic Parameters for GSK3050002 from Male and Female Cynomolgus Monkeys Following Subcutaneous or Intravenous (Slow Bolus) Administration of GSK3050002

| **Parameter** | **Period** |  | **Subcutaneous dose of GSK3050002 (mg/kg/wk)** | | **Intravenous dose of GSK3050002 (mg/kg/wk)** | |
| --- | --- | --- | --- | --- | --- | --- |
|  |  |  | **30 (n=12)** | **300 (n=8)** | **30 (n=12)** | **300 (n=8)** |
| AUC_0-168_  (mg.h / mL) | Week 1 | **Mean** | **30.0** | **187** | **42.2** | **239** |
|  |  | Min | 25.4 | 117 | 37.4 | 160 |
|  |  | Max | 39.9 | 226 | 47.0 | 345 |
|  | Week 13 | **Mean** | **60.1^a^** | **252** | **54.3** | **306** |
|  |  | Min | 37.6^a^ | 179 | 26.8 | 178 |
|  |  | Max | 95.0^a^ | 338 | 65.8 | 397 |
|  | Week 26 | **Mean** | **64.4^a^** | **236^b^** | **55.8^c^** | **311** |
|  |  | Min | 38.2^a^ | 155^b^ | 43.1^c^ | 188 |
|  |  | Max | 105^a^ | 283^b^ | 66.6^c^ | 401 |
| C_max_  (mg/mL) | Week 1 | **Mean** | **0.286** | **2.21** | **0.819** | **7.23** |
|  |  | Min | 0.219 | 1.34 | 0.671 | 6.06 |
|  |  | Max | 0.390 | 3.15 | 0.954 | 9.54 |
|  | Week 13 | **Mean** | **0.517^a^** | **2.35** | **0.912** | **8.17** |
|  |  | Min | 0.363^a^ | 2.09 | 0.760 | 6.31 |
|  |  | Max | 0.736^a^ | 2.82 | 1.05 | 10.6 |
|  | Week 26 | **Mean** | **0.551^a^** | **2.27^b^** | **0.883^c^** | **8.44** |
|  |  | Min | 0.343^a^ | 1.77^b^ | 0.747^c^ | 6.62 |
|  |  | Max | 0.888^a^ | 2.83^b^ | 1.11^c^ | 10.5 |

1. n=11; One female was excluded from mean/median toxicokinetic parameter calculations due to overall lower concentrations across entire profile
2. n=7, One female was excluded from mean/median toxicokinetic parameter calculations due to sharp decline in serum concentrations
3. n=10, One male and one female were excluded from mean/median toxicokinetic parameter calculations due to sharp decline in serum concentrations
