# Supplementary material for: Immune complex disease in a chronic monkey study with a humanised, therapeutic antibody against CCL20 is associated with complement-containing drug aggregates
Source: PLoS One. 2020 Apr 23;15(4):e0231655. doi: 10.1371/journal.pone.0231655 (PMC7180069; doi:10.1371/journal.pone.0231655)
Supplement: S2 Table — (DOCX) [file pone.0231655.s006.docx]

Supplementary Table S2. Summary of histopathological intracellular and extracellular amorphous to crystalline material findings

|  | Males | | | | Females | | | |
| --- | --- | --- | --- | --- | --- | --- | --- | --- |
| Group | Animal # | IHC results | Tissue | Affected injection sites | Animal # | IHC results | Tissue | Affected injection sites |
| Vehicle  (IV and SC) | 1360 | -- |  |  | 1860 | -- |  |  |
|  | 1361 | -- |  |  | 1861 | Extracellular material | Skin (injection site) | 1/4 |
|  | 1362 | Intracellular material | Skin (injection site) | 2/4 | 1862 | -- |  |  |
|  | 1363 | -- |  |  | 1863 | -- |  |  |
|  | 1364-R | -- |  |  | 1864-R | -- |  |  |
|  | 1365-R | -- |  |  | 1865-R | -- |  |  |
| 30 mg/kg/week SC | 2360 | -- |  |  | 2860 | -- |  |  |
|  | 2361 | Intracellular material | Skin (injection site) | 1/4 | 2861 | Intracellular material | Skin (injection site) | 1/4 |
|  | 2362 | Extracellular material | Skin (injection site) | 3/4 | 2862 | -- |  |  |
|  | 2363 | -- |  |  | 2863 | -- |  |  |
|  | 2364-R | -- |  |  | 2864-R | -- |  |  |
|  | 2365-R | -- |  |  | 2865-R | -- |  |  |
| 300 mg/kg/week SC | 3360 | Intracellular material | Skin (injection site) | 3/4 | 3860 | Intracellular material | Skin (injection site) | 1/4 |
|  | 3361 | Intracellular material | Skin (injection site) | 2/4 | 3861 | Intracellular material | Skin (injection site) | 1/4 |
|  | 3362 | Intracellular material | Skin (injection site) | 1/4 | 3862 | Intracellular material | Skin (injection site) | 2/4 |
|  | 3363 | Intracellular material | Skin (injection site) | 2/4 | 3863 | Intracellular material | Skin (injection site) | 1/4 |
| 30 mg/kg/week IV | 4360 | -- |  |  | 4860 | -- |  |  |
|  | 4361 | -- |  |  | 4861 | -- |  |  |
|  | 4362 | -- |  |  | 4862 | -- |  |  |
|  | 4363 | -- |  |  | 4863 | -- |  |  |
|  | 4364-R | -- |  |  | 4864-R | -- |  |  |
|  | 4365-R | -- |  |  | 4865-R | -- |  |  |
| 300 mg/kg/week IV | 5360 | -- |  |  | 5860 | Intracellular material | Liver | NA |
|  | 5361 | -- |  |  | 5861 | -- |  |  |
|  | 5362 | Intracellular material | Esophagus | NA | 5862 | -- |  |  |
|  | 5363 | -- |  |  | 5863 | Intracellular material | Kidney | NA |

IHC = immunohistochemistry; R (shaded) = off-dose animal; -- = no findings; NA = Not Applicable as only 1 tissue sample from these animals was examined.
